# Evolving landscape of female cancers along with attributable risk factors in China from 1990 to 2021, and projections to 2040

**DOI:** 10.3389/fpubh.2025.1629081

**Published:** 2025-11-12

**Authors:** Yali Han, Hui Chen, Didi Song, Rongrong Li, Tongchao Zhang, Xiaorong Yang

**Affiliations:** 1 Department of Radiation Oncology, Qilu Hospital, Cheeloo College of Medicine, Shandong University, Jinan, Shandong, China; 2 Clinical Epidemiology Unit, Qilu Hospital of Shandong University, Jinan, Shandong, China; 3 Clinical Research Center of Shandong University, Qilu Hospital, Cheeloo College of Medicine, Shandong University, Jinan, Shandong, China; 4 Department of Oncology, Zibo Central Hospital, Zibo, Shandong, China; 5 Department of Obstetrics and Gynecology, Qilu Hospital of Shandong University, Jinan, Shandong, China

**Keywords:** female cancers, China, trends, risk factors, projections

## Abstract

**Background:**

Female cancers pose a significant health burden in China, and this study identified and projected epidemiological trends of these cancers.

**Methods:**

We extracted incidence, prevalence, mortality, and disability-adjusted life-years (DALYs) data of female breast, cervical, uterine, and ovarian cancers in China from the Global Burden of Disease (GBD) Study 2021. The estimated annual percentage change (EAPC) and the age-period-cohort (APC) model were used to explore the trends, and the Bayesian APC model (BAPC) was employed to project the burden.

**Results:**

In 2021, breast cancer had the highest incidence (38.58 × 10^4^) and mortality (8.81 × 10^4^) cases, with the age-standardized rates of incidence (ASIR) and prevalence (ASPR) doubling from 1990 to 2021 (EAPC: 2.34 and 2.49). However, the age-standardized rates of mortality (ASMR) and DALYs (ASDR) declined slightly (EAPC: −0.62 and −0.52). Cervical cancer saw a slight increase in ASIR (EAPC: 0.88), a substantial increase in ASPR (EAPC: 2.50), but decreases in ASMR and ASDR (EAPC: −1.05 and −1.07). Uterine and ovarian cancers had slight ASPR increases and moderate ASMR/ASDR declines. High red meat consumption was the leading risk factor for breast cancer, and unsafe sex practices accounted for nearly all cervical cancer DALYs. High BMI contributed to a growing proportion of breast, uterine, and ovarian cancers. Projections indicated increasing burdens for breast, uterine, and ovarian cancers by 2040, with cervical cancer declining.

**Conclusion:**

The female cancer burden in China has been rising and will continue to do so. Targeted prevention and intervention strategies are crucial.

## 1 Introduction

Female cancers, including breast, cervical, uterine, and ovarian cancers, constitute a significant health burden for women worldwide (1). According to the GLOBOCAN 2022 estimates, female breast cancer is the second leading cause of cancer incidence worldwide, with an estimated 2.3 million new cases, the fourth leading cause of cancer mortality, resulting in around 666 thousand deaths globally. Among females, breast cancer is the most frequently diagnosed cancer and the leading cause of cancer deaths globally (2). Cervical cancer ranks fourth in both incidence and mortality among cancers in women, with approximately 660 thousand new cases and 350 thousand deaths worldwide in 2022 (2). These cancers not only impose severe disease burdens on women themselves but also have adverse consequences for their families and offspring. Notably, breast and cervical cancers account for nearly half of the maternal cancer-related orphans (3). Ovarian and uterine cancers exhibit relatively lower incidence rates, but they remain substantial threats to the female reproductive system (4, 5). The burden and trends of female cancers exhibited large geographic and temporal variations across countries and world regions. The socioeconomic development in China has led to significant changes in demographic factors, such as population aging and growth, as well as cancer-related risk factors like environmental influences, lifestyle, and behavior among the Chinese population (6–9). Assessing female cancer burden and risk factors in China is vital to inform trend analysis and guide targeted interventions and resource allocation.

The Global Burden of Diseases (GBD) 2021 study offers a comprehensive dataset to grasp the incidence, prevalence, death, and disability-adjusted life-years (DALYs) of female cancers from 1990 to 2021 in China (10). To our knowledge, this is the first study to comprehensively explore the burden, trends, and risk factors of female cancers across all age groups in China, using the Bayesian age-period-cohort (APC) model to forecast incidence and mortality until 2040. Our findings enhance epidemiological understanding and guide targeted public health policies across prevention, screening, and treatment to improve women’s health and fertility outcomes.

## 2 Materials and methods

### 2.1 Data source and case definition

The comprehensive methodology of the GBD 2021 study has been extensively detailed in previous publications (10–12). Burden data was sourced from the Institute for Health Metrics and Evaluation (IHME). This involved obtaining essential metrics such as counts, rates, and age-standardized rates for incidence (ASIR), prevalence (ASPR), mortality (ASMR), and DALYs (ASDR), from 1990 to 2021. Female cancers were standardized utilizing the International Classification of Diseases (ICD) codes (10). To evaluate the female cancer burden in China, two primary data sources were utilized: surveillance data from the China Disease Surveillance Points (DSP) system and virtual registry (VR) datasets compiled by the Chinese Center for Disease Control and Prevention (CDC) (10). The GBD 2021 study is publicly available with anonymous data; thus, ethics committee review is not required. The flowchart of data source, screening, and statistical analysis was presented in Supplementary Figure S1.

### 2.2 Evaluation of the female cancers burden

The burden of female cancers is typically quantified through the following methods: (1) The original data from two primary data sources underwent a data processing process that mainly includes standardization, mapping data to GBD causes, age/sex splitting, and redistribution (10). (2) Subsequently, two concurrent selection processes were initiated: one aimed at generating input data for the mortality-to-incidence rate (MIR) model, while the other was focused on generating incidence to obtain a final mortality estimate (10). (3) Combined incidence, mortality data, and MIRs to generate mortality estimates. Concurrently, cancer incidence was calculated from cancer mortality estimates using MIRs (10). (4) The cause of death (CoD) database was created, and the years of life lost (YLLs) were assessed using the cause of death ensemble model (CODEm) (10). (5) Based on the GBD incidence estimates and the survival dataset, the final prevalence estimates were evaluated and were split into four sequelae (11). (6) For disability estimation, sequelae-specific years lived with disability (YLDs) were estimated using their specific weights. The disease model meta-regression (DisMod-MR 2.1) was used, and total YLDs were the sum of these sequelae-specific YLDs (11). (7) DALYs were estimated by summing YLLs and YLDs (11).

### 2.3 Attributable risk factors estimation

The GBD risk factor analysis used the comparative risk assessment (CRA) framework to compute estimates, typically involving the following process: (1) For each risk-outcome pair, relative risks (RRs) were individually estimated (12). (2) Estimating exposure levels and distributions of each risk factor using Bayesian models: spatiotemporal Gaussian process regression (ST-GPR) and DisMod-MR 2.1 (12). (3) Based on epidemiological evidence, determine the theoretical minimum risk exposure levels (TMRELs) (12). (4) determined the population attributable fraction (PAF) (12). (5) For each risk, the summary exposure values (SEVs) were computed (12). (6) Mediation factors were assessed to adjust PAF overestimation due to risk factor independence and were used to calculate the burden from multiple risk factor interplay (12). (7) Multiplying PAFs by DALYs for a specific outcome yields measures of attributable burden (12, 13).

### 2.4 Statistical analysis

Data was described with absolute numbers, rates, and 95% uncertainty intervals (UIs). The UIs were derived by utilizing the 2.5th and 97.5th percentiles from a distribution comprising 1,000 draws for each respective metric (11). To assess temporal trends, we utilized the estimated annual percentage change (EAPC) as a metric (14, 15). This was derived using a regression formula: ln(age-standardized rate) = α + β × year + ε. Using this formula, we computed the EAPC and its corresponding 95% confidence interval (CI) via the expression [100 × (exp(β) − 1)] (14, 15).

We utilized a framework based on the APC model to explore potential trends in the burden of female cancer, taking into account factors such as age, period, and birth cohort (16), via the online tool at http://analysistools.nci.nih.gov/apc/. In forecasting the disease burden of female cancers, we employed the Bayesian APC (BAPC) model for predictive analysis, projecting the disease burden up to 2040. (17). This model is formulated as N_ij_ = log(λ_ij_) = μ + α_i_ + β_j_ + γ_k_, where λ_ij_ represents the case count, μ is the intercept, and α_i_, β_j_, and γ_k_, respectively, indicate the influences of age, period, and cohort (17). To address overdispersion, we implemented the BAPC model through the INLA and BAPC packages in R software (17).

Statistical analyses were done using R software (version 4.4.0), and results were visualized, with significance determined at a two-tailed *p*-value threshold of < 0.05.

## 3 Results

### 3.1 The current burden of female cancer in China

In 2021, the estimated number of newly diagnosed female breast cancer patients in China was 38.58 × 10^4^, corresponding to ASIR with around 37.00 per 100,000 population. The number of prevalence cases and ASPR of female breast cancer were 374.81 × 10^4^ and 355.72 per 100,000. Breast cancer contributed to 8.81 × 10^4^ deaths among the female population, and ASMR was 8.24 per 100,000. An estimated 292.11 × 10^4^ DALYs in 2021 were attributed to female breast cancer, with the ASDR at 281.54 per 100,000 population (Table 1).

**Table 1:** Overall disease burden of female breast cancer, cervical cancer, uterine cancer, and ovarian cancer in China from 1990 to 2021.

Indicator	Female breast cancer	Cervical cancer	Uterine cancer	Ovarian cancer
Incidence				
1990: No. × 10^4^	8.48 (6.84, 10.32)	5.78 (4.63, 7.14)	2.63 (1.81, 3.33)	2.00 (1.41, 2.62)
1990: ASIR per 10^5^	17.84 (14.48, 21.65)	11.80 (9.48, 14.54)	5.64 (3.94, 7.10)	4.08 (2.97, 5.31)
2021: No. × 10^4^	38.58 (29.41, 48.90)	13.28 (9.60, 17.26)	7.20 (5.33, 10.00)	4.12 (3.03, 5.45)
2021: ASIR per 10^5^	37.00 (28.23, 46.95)	13.37 (9.61, 17.51)	6.65 (4.91, 9.25)	4.05 (2.96, 5.38)
1990–2021: EAPC	2.34 (2.26, 2.43)	0.88 (0.69, 1.07)	0.40 (0.07, 0.73)	−0.44 (−0.60, −0.29)
Prevalence				
1990: No. × 10^4^	81.91 (68.53, 97.39)	23.67 (18.77, 29.48)	16.31 (11.04, 20.74)	8.26 (5.43, 10.98)
1990: ASPR per 10^5^	175.04 (147.34, 206.19)	44.62 (35.43, 55.49)	33.79 (23.16, 42.75)	15.64 (10.62, 20.74)
2021: No. × 10^4^	374.81 (303.77, 459.93)	74.65 (53.29, 98.31)	50.07 (36.68, 69.87)	17.82 (12.89, 23.69)
2021: ASPR per 10^5^	355.72 (287.01, 437.27)	79.03 (56.34, 103.91)	46.52 (33.62, 64.67)	18.67 (13.57, 24.92)
1990–2021: EAPC	2.49 (2.38, 2.59)	2.50 (2.27, 2.73)	0.94 (0.57, 1.31)	0.27 (0.16, 0.39)
Deaths				
1990: No. × 10^4^	4.04 (3.29, 4.92)	3.18 (2.58, 3.91)	1.07 (0.75, 1.33)	1.18 (0.90, 1.52)
1990: ASMR per 10^5^	8.98 (7.35, 10.88)	6.98 (5.70, 8.56)	2.42 (1.74, 3.01)	2.62 (2.02, 3.35)
2021: No. × 10^4^	8.81 (6.82, 11.03)	4.98 (3.69, 6.44)	1.36 (0.99, 1.86)	2.51 (1.85, 3.29)
2021: ASMR per 10^5^	8.24 (6.37, 10.33)	4.64 (3.44, 6.00)	1.24 (0.91, 1.70)	2.30 (1.70, 3.02)
1990–2021: EAPC	−0.62 (−0.75, −0.48)	−1.05 (−1.21, −0.89)	−2.57 (−2.92, −2.21)	−0.97 (−1.16, −0.78)
DALYs				
1990: No. × 10^4^	146.65 (117.75, 179.80)	111.62 (89.43, 138.10)	34.54 (22.79, 43.81)	40.20 (28.86, 52.71)
1990: ASDR per 10^5^	301.68 (243.17, 368.76)	228.22 (183.51, 282.00)	73.01 (49.39, 91.90)	82.98 (60.96, 108.14)
2021: No. × 10^4^	292.11 (225.45, 371.67)	154.78 (111.98, 200.92)	40.55 (30.11, 55.32)	75.05 (54.58, 99.11)
2021: ASDR per 10^5^	281.54 (216.87, 358.11)	149.84 (108.94, 195.58)	37.86 (28.14, 51.76)	71.16 (51.75, 94.42)
1990–2021: EAPC	−0.52 (−0.64, −0.40)	−1.07 (−1.22, −0.91)	−2.50 (−2.84, −2.16)	−0.99 (−1.17, −0.81)

Data in parentheses are 95% uncertainty intervals (UI) or 95% confidence intervals (CI). ASIR, age-standardized incidence rate; EAPC, estimated annual percentage change; ASPR, age-standardized prevalence rate; ASMR, age-standardized mortality rate; DALYs, disability-adjusted life years; ASDR, age-standardized DALY rates.

The number of incidence cases and ASIR of cervical cancer in 2021 were 13.28 × 10^4^ and 13.37 per 100,000 among the female population. The prevalence of cervical cancer is estimated to be 74.65 × 10^4^ with an ASPR of 79.03 per 100,000. About 4.98 × 10^4^ people died of cervical cancer in 2021, accompanied by ASMR with around 4.64 per 100,000. Cervical cancer caused 154.78 × 10^4^ DALYs, and ASDR was 149.84 per 100,000 (Table 1).

The estimated number of new cases of uterine cancer was 7.20 × 10^4^ with an ASIR of 6.65 per 100,000. The prevalence of uterine cancer was reported to be 50.07 × 10^4^ cases with an ASPR of 46.52 per 100,000. Uterine cancer contributed to 1.36 × 10^4^ deaths, and ASMR was 1.24 per 100,000. Additionally, uterine cancer resulted in 40.55 × 10^4^ DALYs with an ASDR of 37.86 per 100,000 (Table 1).

There were an estimated 4.12 × 10^4^ new cases of ovarian cancer, with an ASIR of 4.05 per 100,000. The prevalence of ovarian cancer was estimated to be 17.82 × 10^4^ cases with an ASPR of 18.67 per 100,000. Ovarian cancer contributed to 2.51 × 10^4^ deaths, and ASMR was 2.30 per 100,000. Besides, an estimated 75.05 × 10^4^ DALYs were attributed to ovarian cancer, with an ASDR of 71.16 per 100,000 (Table 1).

**Figure 1:**
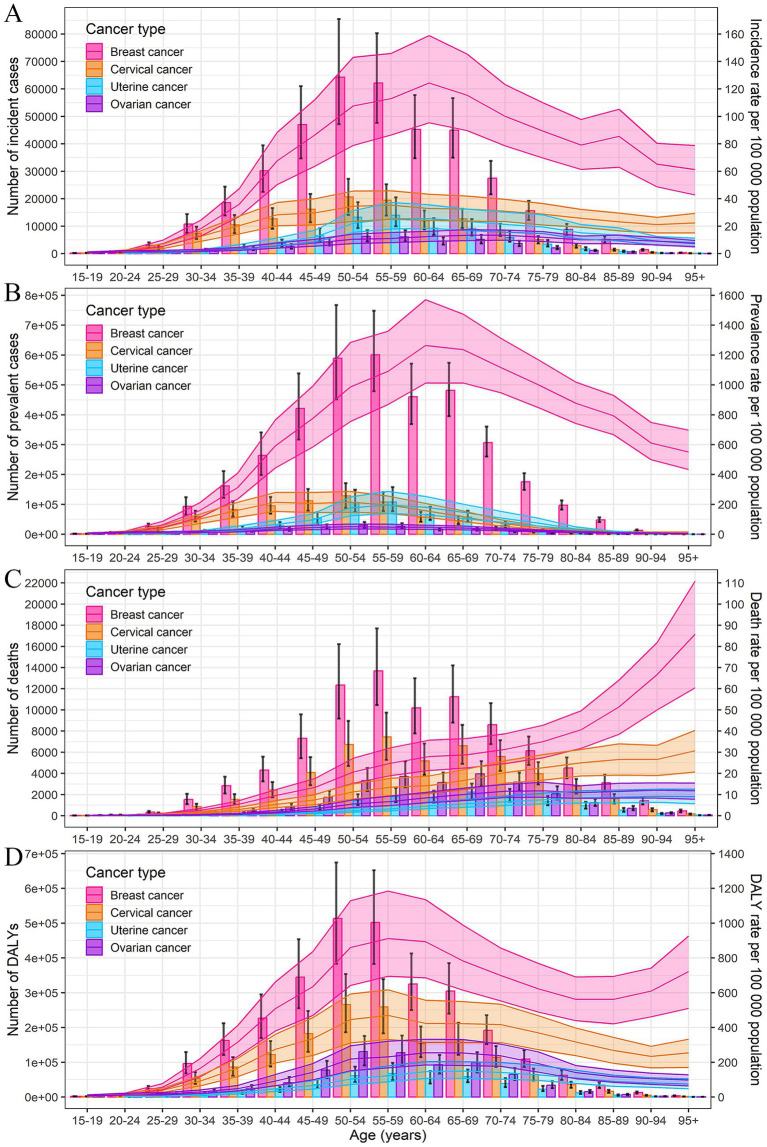
Numbers and rates of incidence **(A)**, prevalence **(B)**, deaths **(C)**, and DALYs **(D)** of female breast, cervical, uterine, and ovarian cancer by age in 2021 in China. Error bar represents the upper and lower limits of the 95% uncertainty intervals (95% UIs) of the number. Shading represents the upper and lower limits of the 95% uncertainty intervals (95% UIs) of rate. DALYs, disability-adjusted life-years.

The incidence rates for breast and uterine cancer peaked in the 60–64 age group, cervical cancer in the 55–59 age group, and ovarian cancer in the 65–74 age group. The prevalence rates for breast cancer peaked at 60–64 years, cervical and ovarian cancer at 50–54 years, and uterine cancer at 55–59 years. The highest mortality rates were observed in those over 95 years old for breast and cervical cancer, and in the 90–94 age group for uterine and ovarian cancer. The peak DALYs rates were seen at 55–59 years for breast and cervical cancer, 65–69 years for uterine cancer, and 60–64 years for ovarian cancer (Figure 1; Supplementary Table S1). Overall, the diagnoses of female cancers predominantly occurred between the ages of 55–74 years, with the highest burden of DALYs rates observed in the ages of 55–69 years.

### 3.2 Trends of female cancer burden from 1990 to 2021

**Figure 2:**
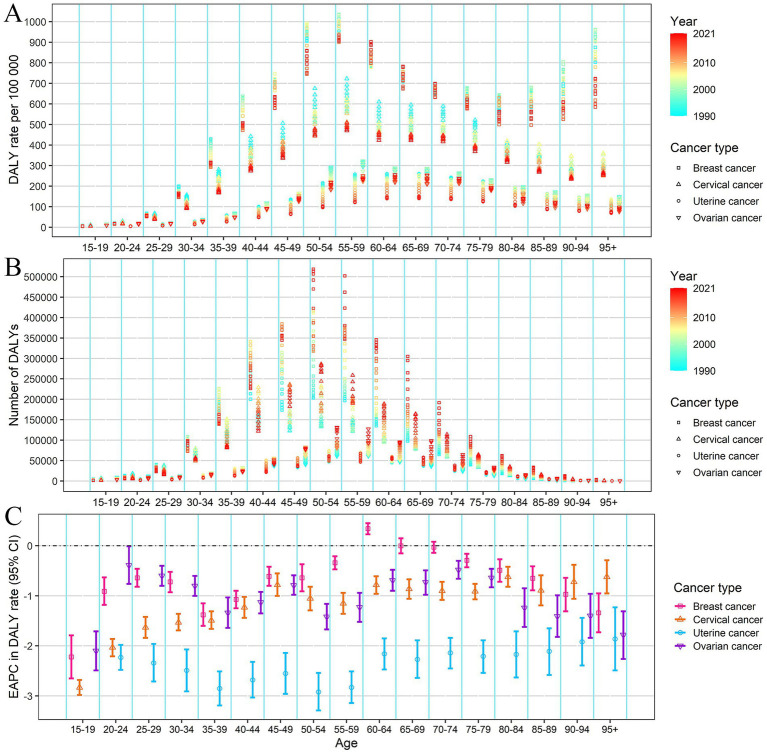
DALYs rate **(A)**, number of DALYs **(B)** of female cancers by age, from 1990 to 2021 in China; EAPC of DALYs rate **(C)** of female cancer by age in China. Error bar represents the upper and lower limits of the 95% confidence interval (95% CIs) of EAPC. EAPC, estimated annual percentage change. DALYs, disability-adjusted life-years.

From 1990 to 2021, the numbers of incidence, prevalence, mortality, and DALYs of breast cancer significantly increased among the female population. The ASIR and ASPR of breast cancer doubled from 1990 to 2021 (EAPC = 2.34, and 2.49, respectively) (Supplementary Figure S2; Table 1). The increasing trend was observed across most age groups, with the most pronounced increase in the 60–64 age group (Supplementary Figures S4, S5; Supplementary Table S1). The ASMR and ASDR for breast cancer present an overall declining trend (EAPC = −0.62, and −0.52, respectively), but exhibited an upward trend from 2014 to 2021 (Supplementary Figure S2). The decline in DALYs rate and mortality rate from 1990 to 2021 was observed across all age groups, except for the 60–64 age group (Figure 2 and Supplementary Figure S3; Supplementary Table S1).

For cervical, uterine, and ovarian cancer, the numbers of incidence, prevalence, mortality, and DALYs increased slowly from 1990 to 2021. The ASIR of cervical cancer (EAPC = 0.88) and uterine cancer (EAPC = 0.40) all presented a slight increasing trend, while the ASIR of ovarian cancer showed a slight declining trend (EAPC = −0.44) (Table 1; Supplementary Figure S2). Notably, the incidence rates for ovarian cancer declined in most age groups, except for an increase noted in the 20–34 age group (Supplementary Figure S4; Supplementary Table S1). The mortality and DALYs rates of cervical, uterine, and ovarian cancer showed a consistent downward trend across all age groups (Figure 2 and Supplementary Figures S2, S3).

### 3.3 Age, period, and cohort effects in female cancer

**Figure 3:**
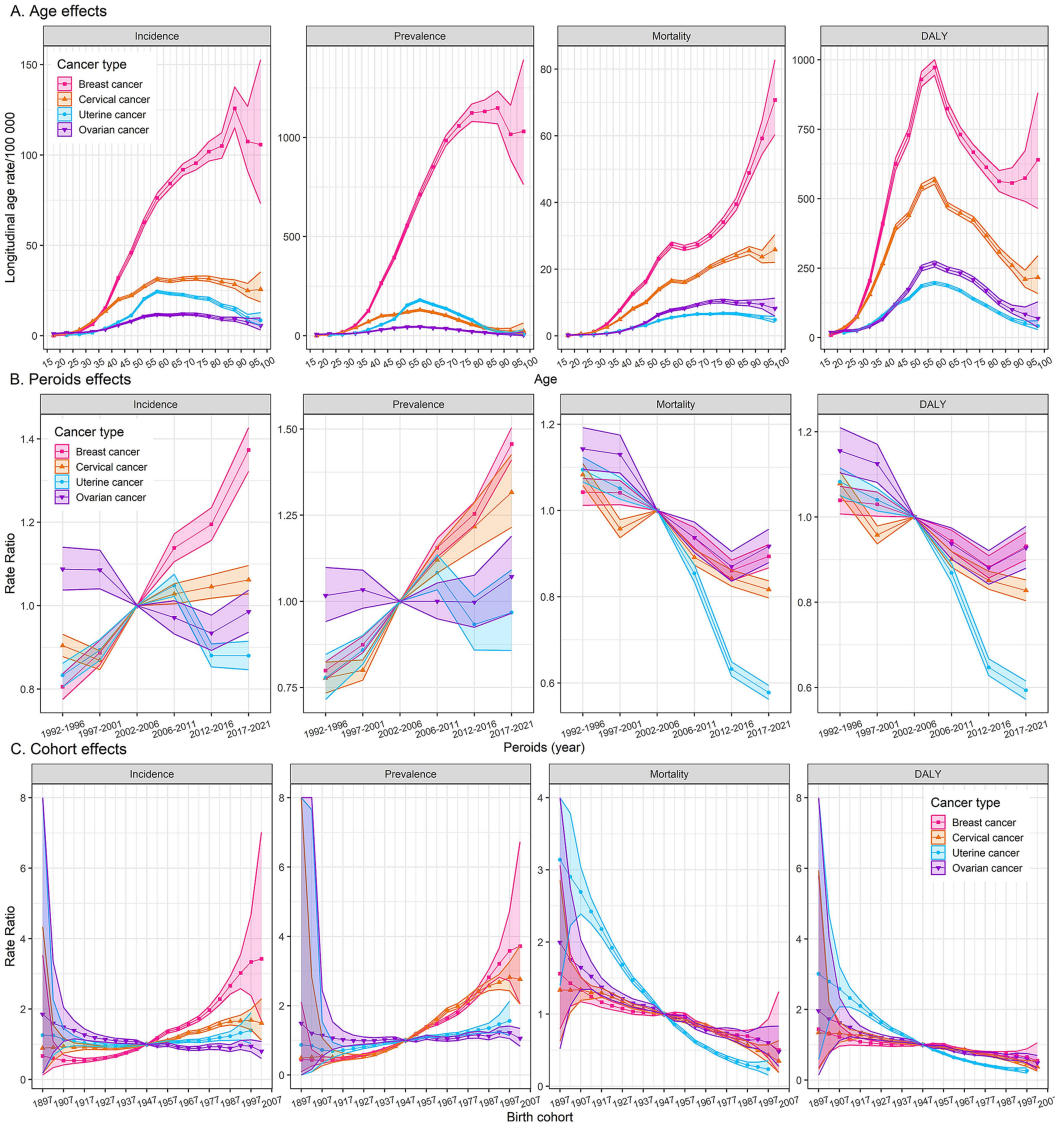
Age, period, and cohort effects on female cancers burden. Fitted longitudinal age curves of incidence, prevalence, mortality, and DALYs (per 100,000 person-years) **(A)**. The relative risk of each period adjusted for age and nonlinear cohort effects **(B)**. The relative risk of each cohort adjusted for age and nonlinear period effects **(C)**. The dots and shaded areas denote rates or rate ratios and their corresponding 95% confidence intervals (95% CIs). DALYs, disability-adjusted life-years.

Age effects of the incidence and prevalence rates for breast cancer exhibit a sharp increase with age, particularly after the age of 40, peaking in the 85–89 age group, followed by a decline and fluctuation. Age effects in the mortality rate of breast cancer showed a linear increase, and the rates surged after age 85. The DALYs rate for breast cancer increases with age, peaking in the 55–60 age group, then declining until age 90, followed by a slow rise. As for cervical, uterine, and ovarian cancer, the incidence and prevalence rates gradually increase with age, peaking in the 55–60 age group, and then decreasing slowly. The mortality rate of cervical cancer shows a linear increase with age, while the mortality rates for uterine and ovarian cancer show a gradual increase with age, followed by a slight decrease after age 80. The DALYs rates of cervical, uterine, and ovarian cancer all peak in the middle-aged group of 55–60, with a subsequent decline (Figure 3A).

Period effects generally showed a significantly increasing risk of incidence and prevalence for breast cancer over the study period, while a generally declining risk of mortality and DALYs, with a notable resurgence post-2012. Cervical cancer experienced its lowest period risk of incidence between 1997 and 2001, followed by a gradual increase. Conversely, mortality and DALYs rates for cervical cancer showed a significant decline after the period of 2002–2006. The incidence and prevalence rates of uterine cancer reached their peak in the period of 2006–2011, while the mortality and DALYs rates exhibited a clear downward trend over the study period. Period effects on the incidence, mortality, and DALYs for ovarian cancer displayed a downward trend after the period of 1997–2001, followed by a resurgence post the period of 2012–2016 (Figure 3B).

Cohort analyses reveal unfavorable trends in the incidence and prevalence of breast cancer among more recent cohorts, while a gradually declining risk of mortality and DALYs. The incidence and prevalence rates of cervical cancer are elevated in older cohorts but have decreased in more recent ones, indicating a possible success in prevention or early detection. Unlikely, the incidence and prevalence rates for uterine and ovarian cancer have remained relatively stable across all birth cohorts. Cohort effects on mortality and DALYs rates for cervical cancer and ovarian cancer followed similar declining trends observed in breast cancer, while uterine cancer exhibited a more pronounced decrease in recent cohorts (Figure 3C).

### 3.4 Risk factors of female cancer in China

**Figure 4:**
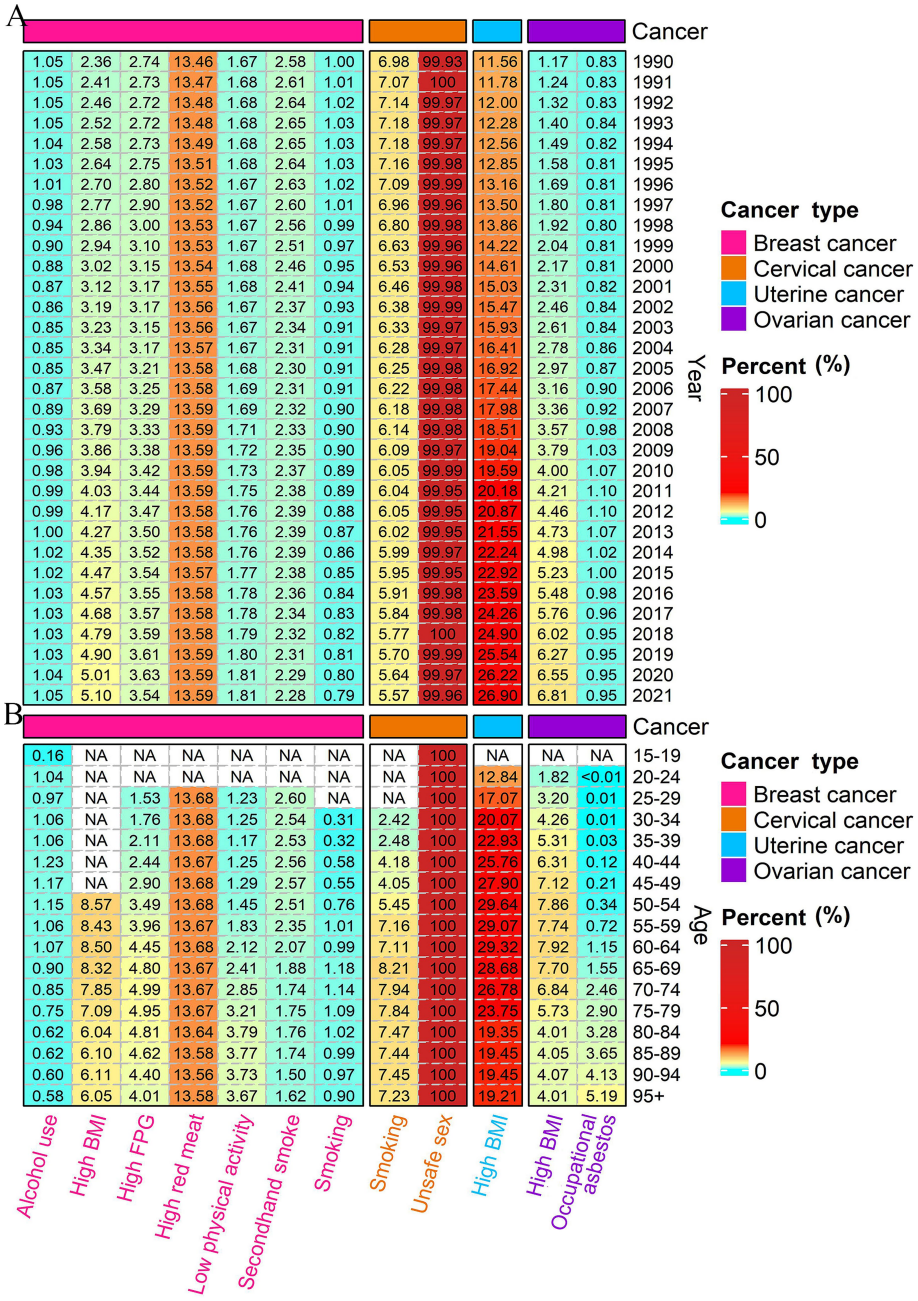
Percentage of DALYs attributable to risk factors from 1990 to 2021 in China **(A)**; and percentage of DALYs attributable to risk factors by age in 2021 in China **(B)**. NA represents a missing value. DALYs, disability-adjusted life-years.

High red meat consumption was identified as the predominant contributor to DALYs of breast cancer, accounting for approximately 13% of the total burden, with a stable contribution across all years and age-specific cohorts (Figure 4). The attributable percentage of DALYs for breast cancer due to high BMI and elevated FPG has shown a gradual increase over the years. Other risk factors, such as alcohol use, low physical activity, secondhand smoke, and smoking, constitute a smaller proportion of the DALYs, remaining generally stable over time but exhibiting variations across different age groups. Notably, low physical activity has a more significant impact on older age populations. Nearly all DALYs for cervical cancer, across all age groups and over the years, were attributable to unsafe sex practices. Additionally, 5–7% of these DALYs were attributed to smoking, particularly in individuals aged 55 years and older. It is noteworthy that the proportion of DALYs for uterine and ovarian cancer attributable to high BMI has increased over the years. For uterine cancer, this proportion rose from 11.56% in 1990 to 26.90% in 2021, and for ovarian cancer, it increased from 1.17% in 1990 to 6.81% in 2021. Besides, the proportion of DALYs for ovarian cancer attributable to occupational asbestos exposure remained stable over the years and was highest in the older population, specifically those over 70 years old (Figure 4). Risk-attributable female cancer mortality analysis revealed consistent results (Supplementary Figure S6).

### 3.5 Predictions of incidence and mortality of female cancer from 2022 to 2040

**Figure 5:**
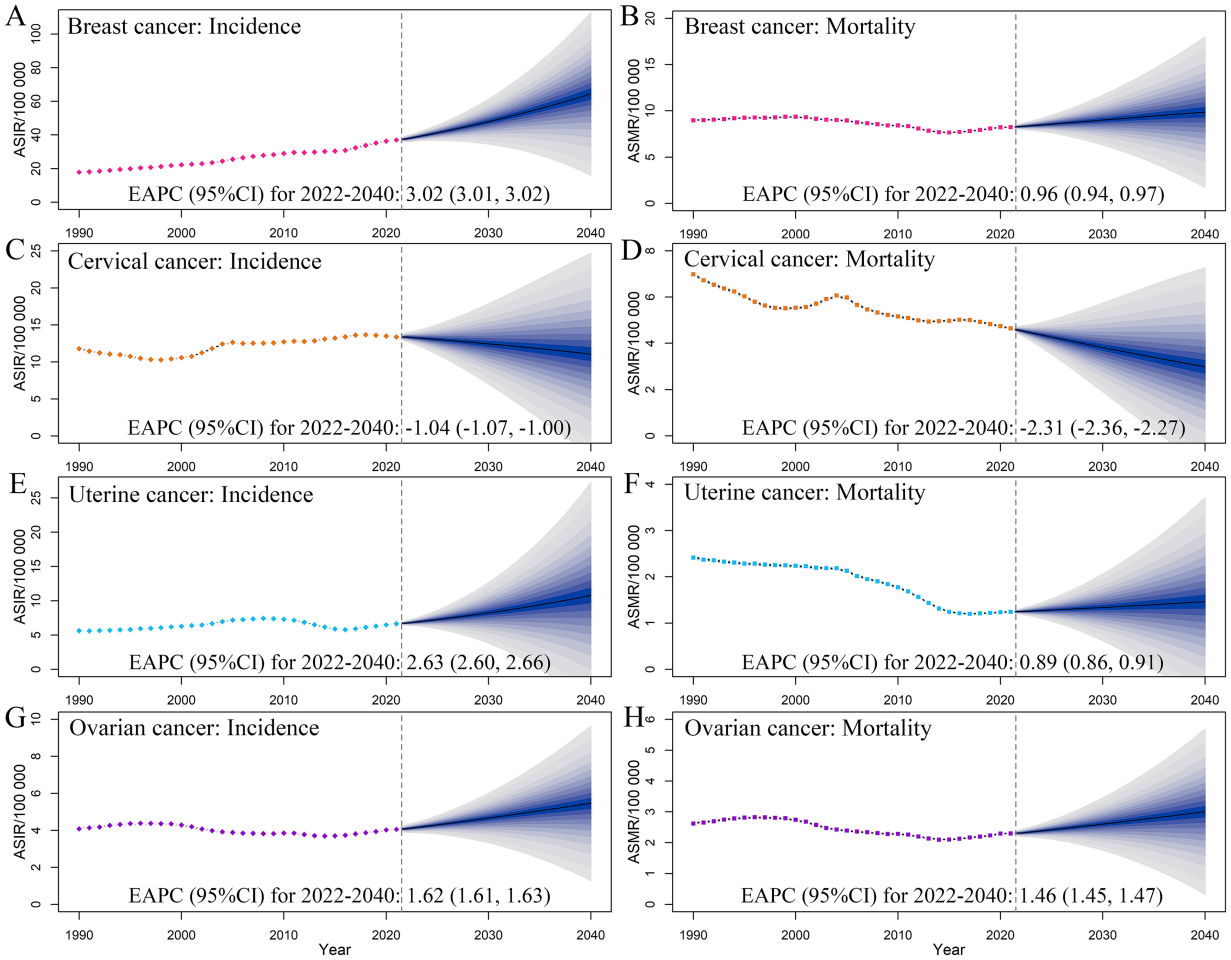
Temporal trends and projected age-standardized rates of incidence **(A,C,E,G)** and mortality **(B,D,F,H)** of female cancers, from 1990 to 2040 in China. Shading represents a 1% decrease and increase interval based on the 2021 rate. EAPC, estimated annual percentage change. CI, confidence interval.

Based on predictions, by 2040, the numbers of female breast cancer incident cases, prevalent cases, deaths, and DALYs would increase to 87.08 × 10^4^, 830.28 × 10^4^, 16.22 × 10^4^, and 472.22 × 10^4^, respectively; the corresponding ASIR, ASPR, ASMR, and ASDR would also increase to 64.38, 604.11, 9.86, and 347.15 per 100,000, respectively (Figure 5; Supplementary Figure S7; Supplementary Table S2). For cervical cancer, the ASIR, ASPR, ASMR, and ASDR would decrease to 11.07, 61.50, 2.98, and 98.50 per 100,000, respectively. For uterine cancer, the ASIR, ASPR, ASMR, and ASDR would also increase to 10.79, 76.55, 1.47, and 43.23 per 100,000, respectively. Similar trends were also identified in ovarian cancer burden, with the ASIR, ASPR, ASMR, and ASDR increasing to 5.46, 25.32, 3.01, and 90.66 per 100,000, respectively (Figure 5; Supplementary Table S2).

## 4 Discussion

In 2021, about 631.8 thousand new cases of breast, cervical, uterine, and ovarian cancers were reported among women in China, leading to about 176.6 thousand deaths and about 5,624.9 thousand DALYs, posing a severe threat to women’s health and lives in China. These numbers are concerning and highlight the urgent need for increased awareness, prevention, and early detection efforts for these cancers among women in China. A previous study analyzed the epidemiological trends of female cancers in adolescents and young adults in China from 1990 to 2019 and revealed that the incidence rate grew continuously, while the mortality rate maintained a steady state in adolescents and young adults in China (18). However, we found that the highest incidence and DALYs rates for female cancers are predominantly in the 55–70 age group. Therefore, it is crucial to conduct a comprehensive analysis of the entire population for a better understanding of the epidemiological trends and implementing targeted public health strategies.

The overall burden of female breast cancer in China is much higher than that of cervical, uterine, and ovarian cancer. From 1990 to 2021, the incidence and prevalence rates showed a significant increasing trend among women under 90 years old. Notably, our findings revealed an overall decreasing mortality and DALYs rates during 2000–2014, probably due to treatment breakthroughs, improved early detection through screening, and increased public awareness. However, the mortality rates for breast cancer increased from 2014 to 2021, potentially related to population aging as indicated by the APC analysis. Moreover, the burden of breast cancer is projected to grow to over 870 thousand new cases and over 162 thousand deaths by 2040. In terms of attributable risk factors for mortality of female breast cancer, high red meat consumption constitutes the predominant contributor across the population. Elevated blood-sugar levels, high BMI, and low physical activity warrant higher attention in middle-aged and older individuals, whereas alcohol and tobacco consumption should be addressed in young adults. Other well-documented risk factors for breast cancer include early menarche, late menopause, advanced age at first birth, fewer children, less breastfeeding, hormone-replacement therapy, and oral contraceptives (2, 19). The World Health Organization (WHO) and the Global Breast Cancer Initiative (GBCI) proposed three key strategies to reduce breast cancer mortality: health promotion and early detection, timely diagnosis, and comprehensive breast cancer management (20). China has been devoted to implementing high-quality, population-based screening programs, with breast and cervical cancer screening being particularly successful in reaching a large number of rural women (21).

Cervical cancer ranks as the second leading cancer among the four cancers in Chinese women in terms of both incidence and mortality (9). The risk of developing cervical cancer in cohorts born after 1997 has decreased, which can be partially attributed to vaccination or early screening. The mortality rate of cervical cancer has generally shown a downward trend, except for a fluctuating increase between 1998 and 2004. Nearly all cervical cancers are mainly triggered by Human Papillomavirus (HPV) infection, with other contributing factors being smoking, multiple childbirths, and oral contraceptives (22). According to current guidelines, HPV vaccination should be initiated for all children, regardless of sex, at the age of 9, with two doses administered before the age of 13, and three doses for those starting at age 15 or older (23, 24). In 2022, the first dose of HPV vaccine coverage for females aged 9–45 in China was 10.15%, while it was just 4.00% for those aged 9–14 (25). This underscores the urgent need to enhance vaccination efforts, particularly among younger adolescents. Besides, it is recommended that individuals aged 25 through 65 years undergo screening at least every 5 years (24, 26). The WHO declared specific goals in 2020: 90% of girls are fully vaccinated with the HPV vaccine by age 15 years; 70% of women are screened with a high-performance test by age 35 years and again by age 45 years; and 90% of women identified with cervical disease receive treatment (27). The National Health Commission (NHC) of China has actively responded to and supported the WHO policies (28). The Chinese government has also launched a series of screening and prevention projects for cervical cancer, including the newly released China Women’s Development Guidelines (2021–2030) and the Healthy China Action Plan (2019–2030), which specified objectives and strategies for improving screening coverage, promoting HPV vaccination, and enhancing the accessibility of HPV vaccines (29). Nevertheless, China still faces many challenges in trying to implement these strategies.

In China, uterine cancer and ovarian cancer present a relatively low burden, but still significantly impact the reproductive health of Chinese women. The observed decrease in overall mortality rates for uterine and ovarian cancers in China reflects significant advancements in medical resources and healthcare quality, including improved access to advanced diagnostic and treatment options, and the implementation of more effective and targeted cancer care strategies. Notably, the incidence rates for ovarian cancer and uterine cancer both increased significantly between the ages of 20 and 34. This highlights the significance of strengthening public health education, promoting healthy lifestyles, and improving early screening for uterine and ovarian cancers. Our research shows that being overweight increases the risk of uterine and ovarian cancer, and the trend has worsened over time. A recent report indicates that from 2015 to 2019, nearly 35% of adults in China were overweight and over 16% were obese (30). Studies link obesity to a higher risk of various cancers and poorer treatment outcomes, driven by pro-inflammatory signaling, hormonal disruptions, and insulin resistance that create a tumor-friendly environment (31). Additionally, obesity in cancer patients leads to worse prognoses due to reduced drug efficacy, increased comorbidities, and surgical challenges (32, 33). These findings highlight the importance of integrating body weight management into cancer treatment strategies. Besides, we and others verified the link between asbestos exposure and ovarian cancer, particularly in the older population, highlighting the importance of sustained efforts to minimize asbestos exposure and protect public health (34). This is especially crucial in light of the well-documented risks of asbestos exposure for other cancers, including mesothelioma and lung cancer (35). In addition, extensive epidemiological research has identified hormonal imbalances, metabolic disorders, diabetes mellitus, and hypertension, as key risk factors for the development of uterine and ovarian cancer (36, 37). China has made significant strides in managing and treating uterine and ovarian cancer, including comprehensive surgical staging, minimally invasive techniques, adjuvant chemotherapy, and radiotherapy. For ovarian cancer, early detection and prevention are crucial, as most patients are diagnosed at advanced stages (38). Genetic screening for BRCA1/2 mutations is recommended for women with a family history (39). Additionally, the combination of serum CA125 and transvaginal ultrasound is being explored for early ovarian cancer screening (40). These advancements are expected to reduce the burden and improve outcomes for these cancers. However, our predictive model indicates that the burden of uterine and ovarian cancer will likely continue to rise over the next 19 years, highlighting continued efforts in disease prevention and control.

Some limitations in this study should be considered. First, the accuracy of our findings relies on the quality of the GBD data, which is sourced from various quality levels. Despite our efforts to minimize bias, potential selection and recall biases may still exist. Second, we could not account for other risk factors specific to certain cancers or age groups, such as genetic and reproductive factors, due to limited information from the GBD data. Third, we did not analyze other female cancers due to space constraints. Fourth, our predictions did not consider crucial factors like changes in China’s socioeconomic structure, population characteristics, and advancements in screening and treatment technologies. Lastly, our burden assessment was limited to the national level and did not include a more detailed evaluation at the provincial level. Therefore, these findings should be interpreted with caution and updated regularly as new data become available.

## 5 Conclusion

In conclusion, female cancers pose a significant challenge in China, with rising incidence and prevalence rates from 1990 to 2021. Notably, cervical cancer mortality and DALY rates have declined, with projected decreases in incidence and mortality over the next 19 years. However, breast, ovarian, and uterine cancers are expected to impose a growing burden. Thus, prevention measures should be strengthened, public awareness raised, and access to medical services in resource-limited areas improved to reduce this burden.

## Data Availability

Publicly available datasets were analyzed in this study. This data can be found at: the GBD 2021 study is a publicly available database (https://vizhub.healthdata.org/gbd-results/), and we fully comply with data usage requirements.

## Glossary

DALYs: disability-adjusted life-years
EAPC: estimated annual percentage change
ASIR: age-standardized incidence rate
ASPR: age-standardized prevalence rate
ASMR: age-standardized mortality rate
ASDR: age-standardized DALYs rate
BMI: body mass index
BAPC: Bayesian age-period-cohort
APC: Age-Period-Cohort
GBD: Global Burden of Diseases
FPG: fasting plasma glucose
IHME: Institute for Health Metrics and Evaluation
ICD: International Classification of Diseases
DSP: Disease Surveillance Points
VR: virtual registry
CDC: Chinese Center for Disease Control and Prevention
MIR: mortality-to-incidence rate
CoD: cause of death
YLLs: years of life lost
CODEm: cause of death ensemble model
SEER: Surveillance, Epidemiology, and End Results Program
YLDs: years lived with disability
DisMod-MR: disease model meta-regression
CRA: comparative risk assessment
RRs: relative risks
ST-GPR: spatiotemporal Gaussian process regression
TMRELs: theoretical minimum risk exposure levels
PAF: population attributable fraction
SEVs: summary exposure values
UIs: uncertainty intervals
CI: confidence interval
WHO: World Health Organization
GBCI: Global Breast Cancer Initiative
NHC: National Health Commission.
