# Supplementary material for: Evolving landscape of female cancers along with attributable risk factors in China from 1990 to 2021, and projections to 2040
Source: Front Public Health. 2025 Nov 12;13:1629081. doi: 10.3389/fpubh.2025.1629081 (PMC12646900; doi:10.3389/fpubh.2025.1629081)
Supplement: Supplementary file 3 [file Data_Sheet_1.pdf]

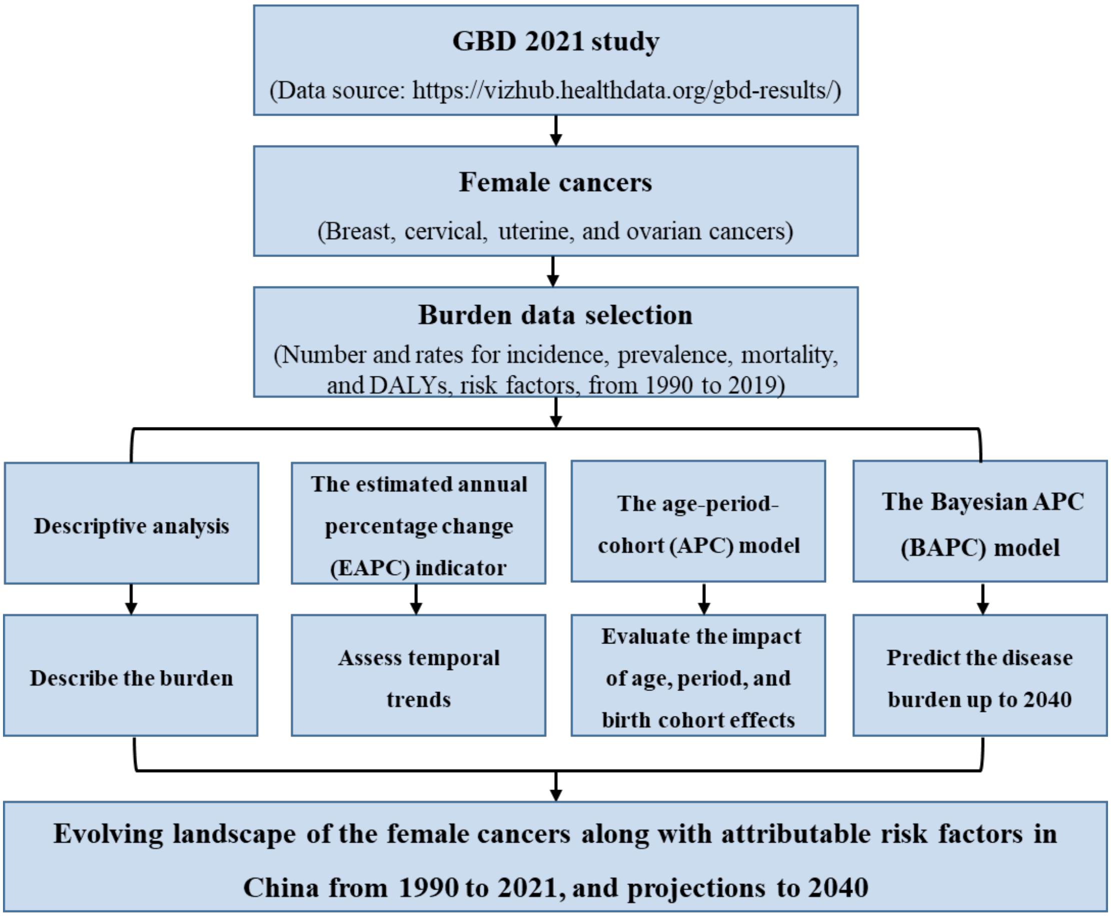

Figure S1. The flowchart of data source, screening, and statistical analysis.

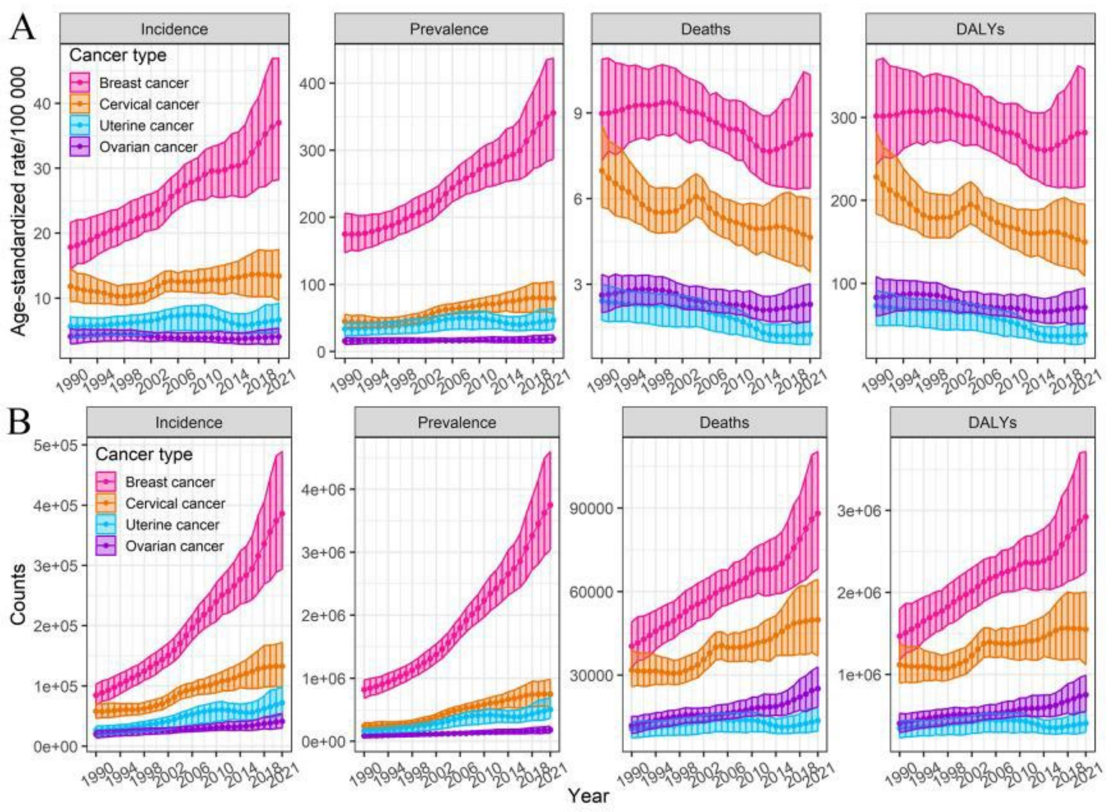

Figure S2. Age-standardized rates (A) and numbers (B) of incidence, prevalence, deaths, and DALYs for female breast, cervical, uterine, and ovarian cancer in China from 1990 to 2021. Shading represents the upper and lower limits of the 95% uncertainty intervals (95% UIs). DALYs, disability-adjusted life-years.

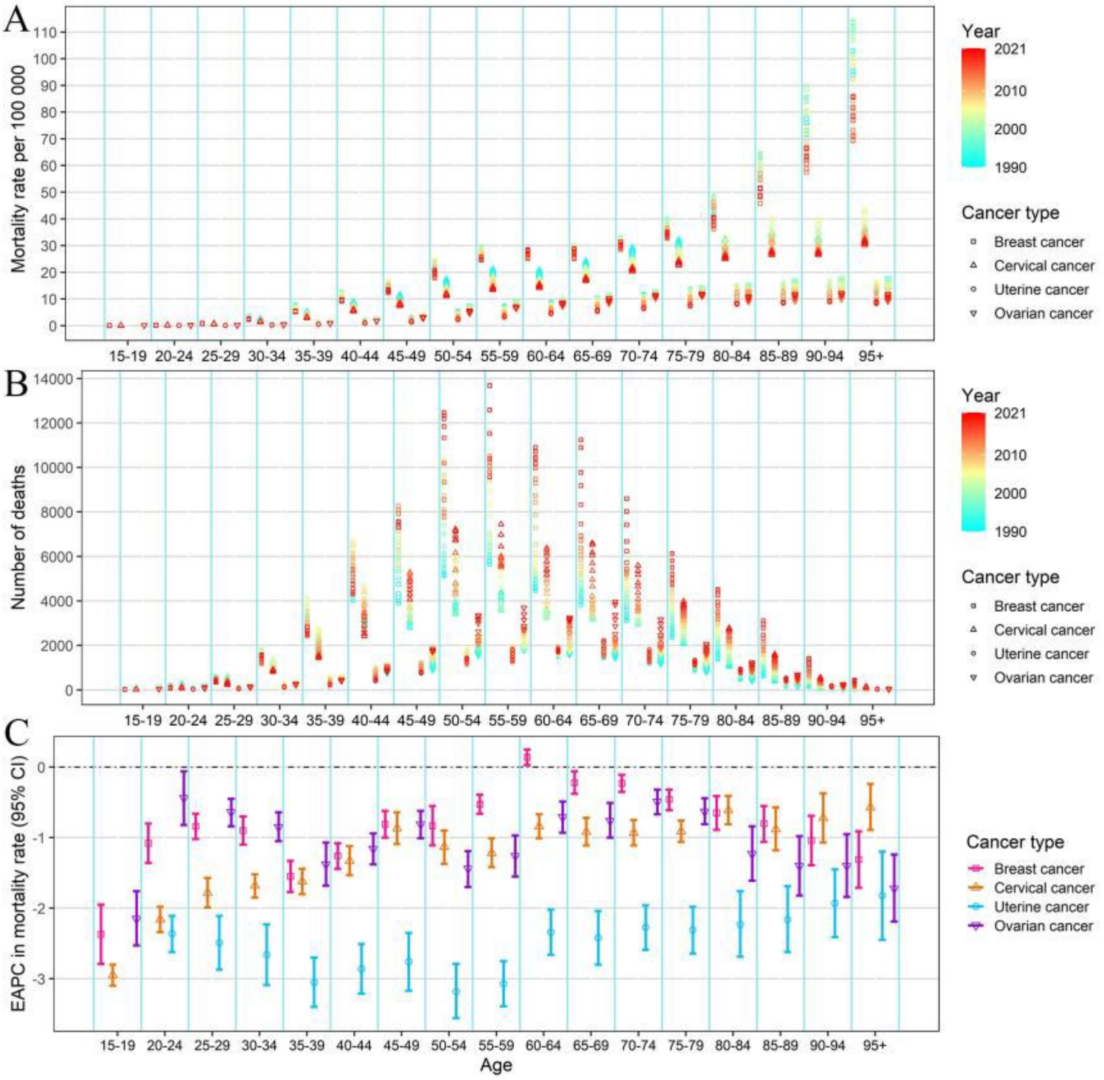

Figure S3. Mortality rate (A), number of deaths (B) of female cancer by age, from 1990 to 2021 in China; EAPC of mortality rate (C) of female cancer by age in China. Error bar represents the upper and lower limits of the 95% confidence interval (95% CIs) of EAPC. EAPC, estimated annual percentage change.

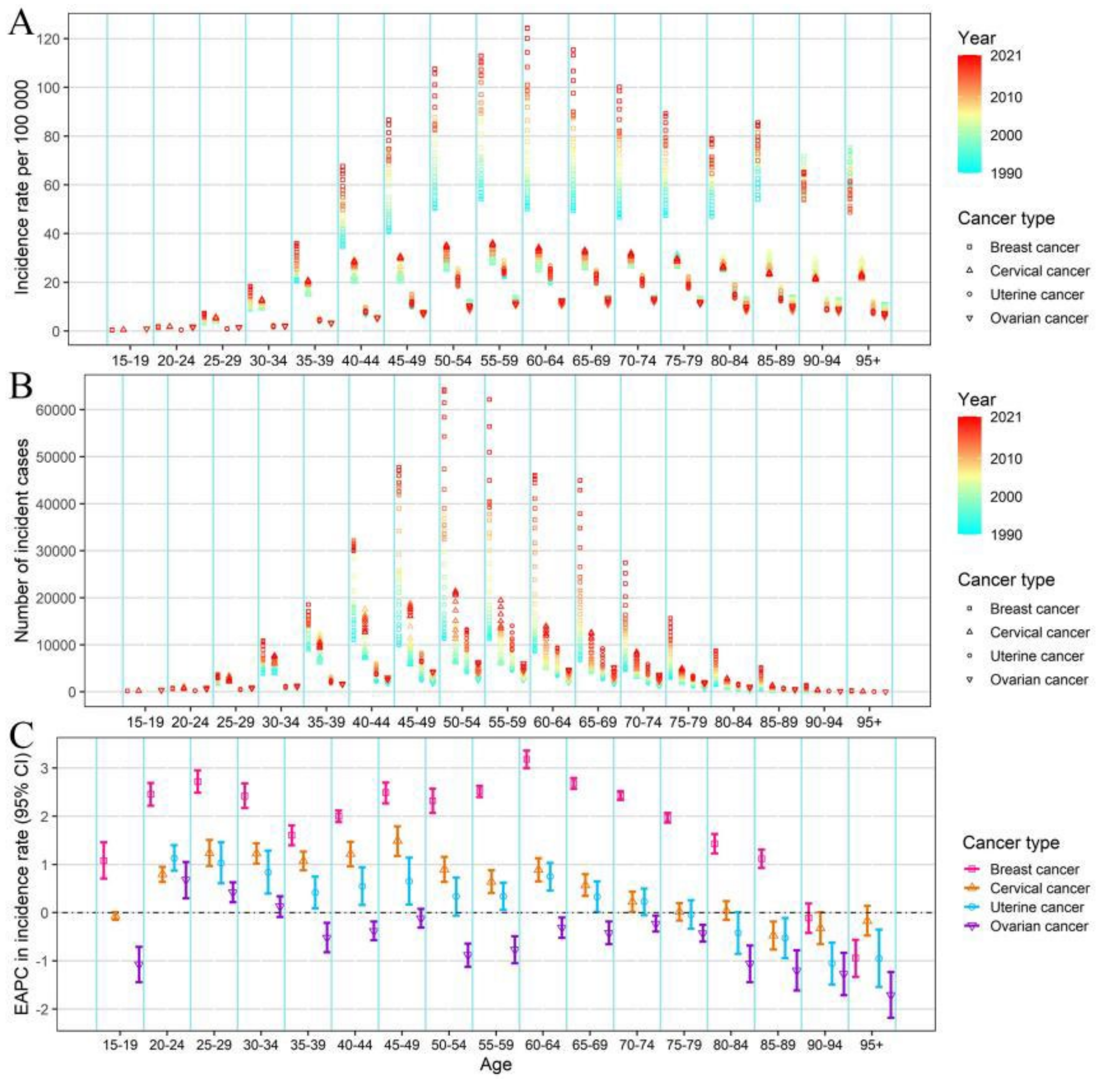

Figure S4. Incidence rate (A), incidence cases (B) of female cancer by age, from 1990 to 2021 in China; EAPC of incidence rate (C) of female cancer by age in China. Error bar represents the upper and lower limits of the 95% confidence interval (95% CIs) of EAPC. EAPC, estimated annual percentage change.

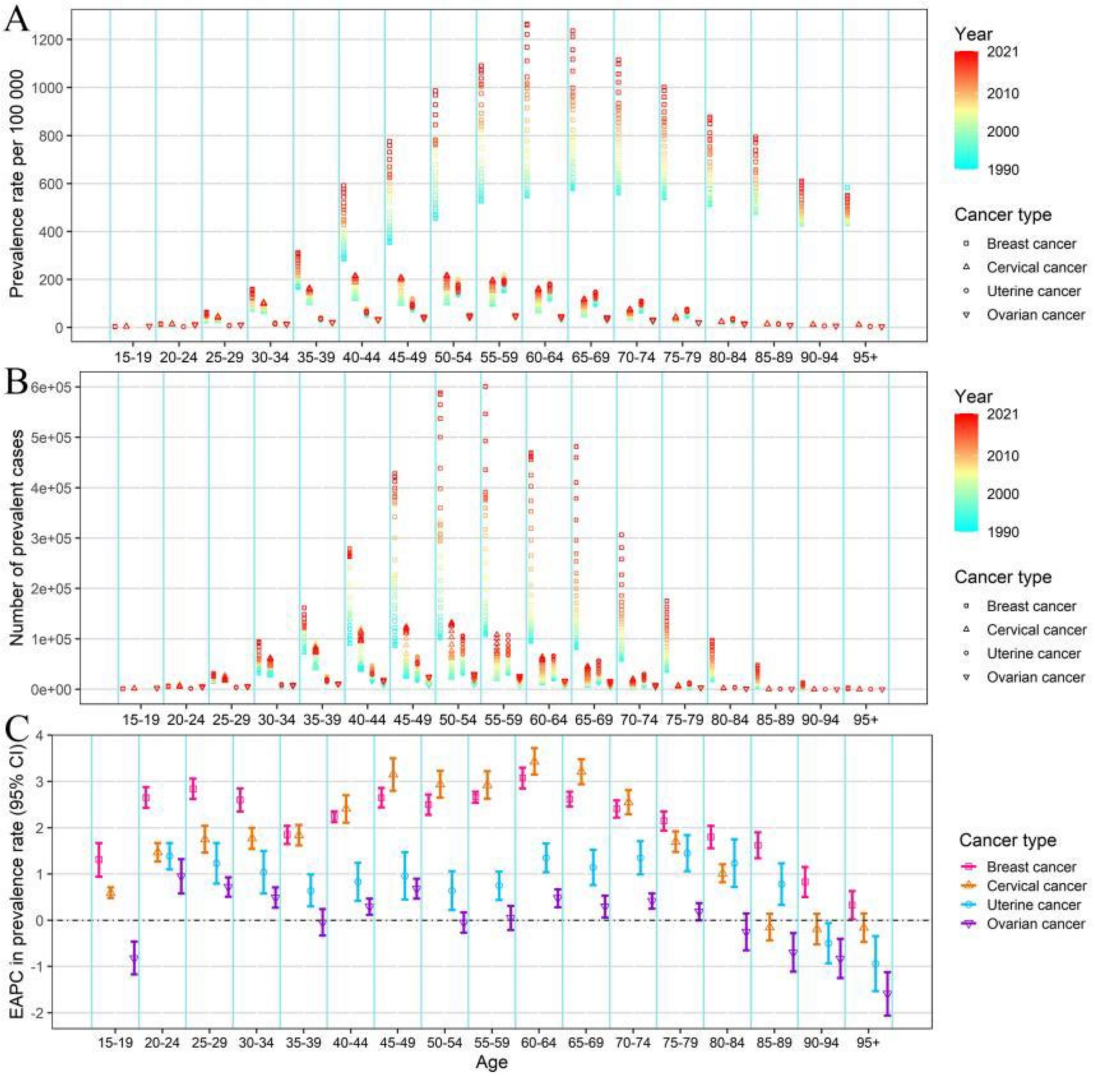

Figure S5. Prevalence rate (A), prevalence cases (B) of female cancer by age, from 1990 to 2021 in China; EAPC of prevalence rate (C) of female cancer by age in China. Error bar represents the upper and lower limits of the 95% confidence interval (95% CIs) of EAPC. EAPC, estimated annual percentage change.

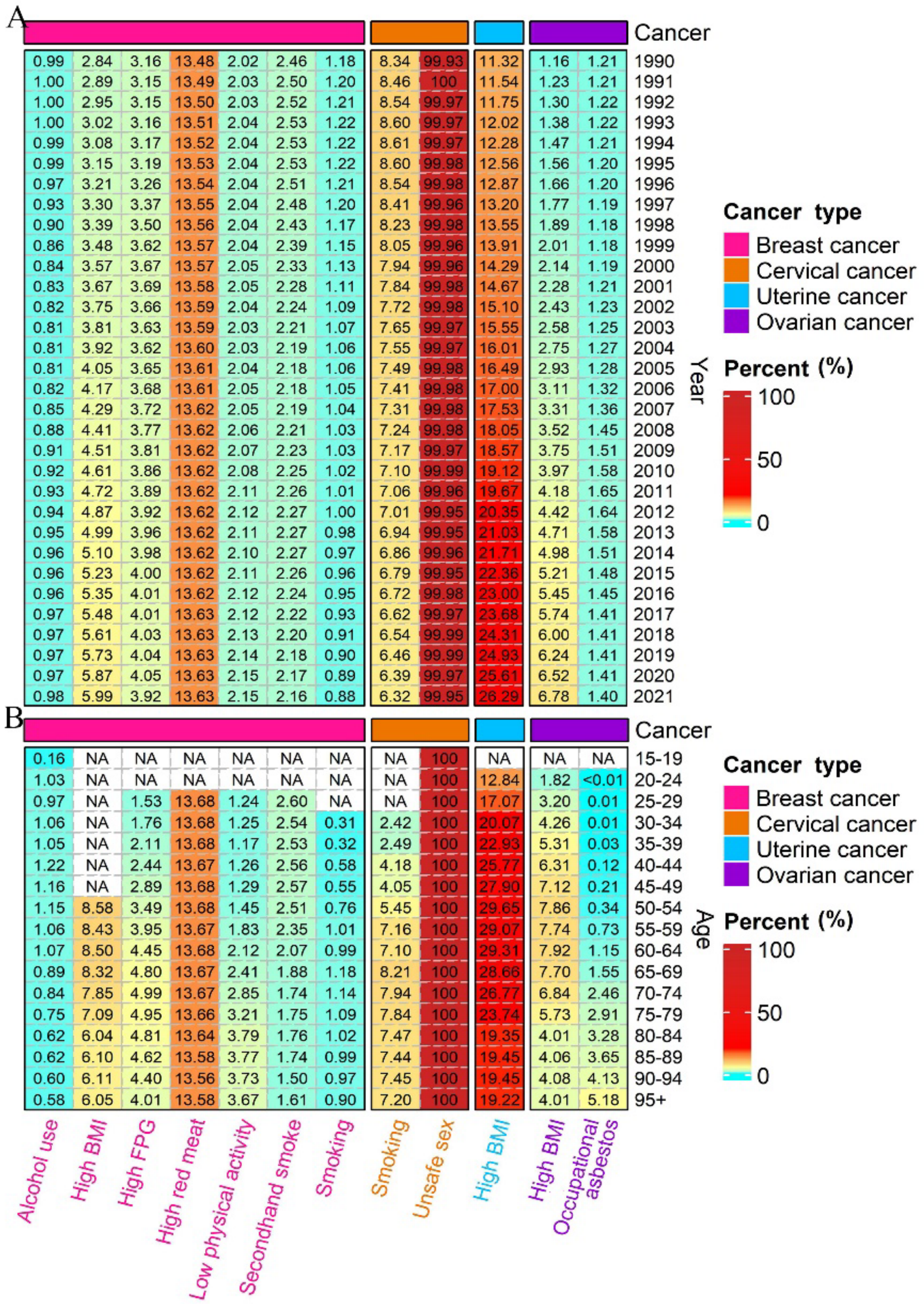

Figure S6. Percentage of mortality attributable to risk factors from 1990 to 2021 in China (A); and percentage of mortality attributable to risk factors by age in 2021 in China (B). NA represents missing value.

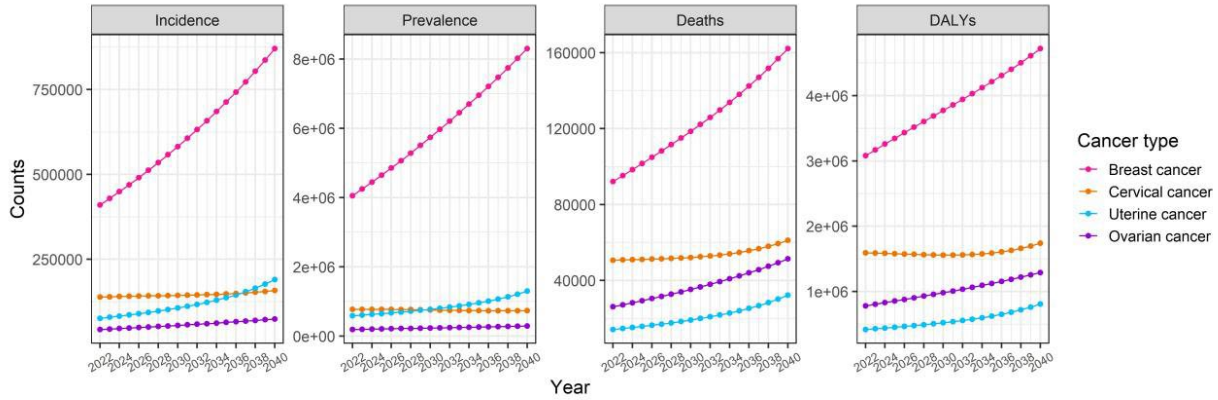

Figure S7. Trends in projected numbers of incidence, prevalence, deaths, and DALYs of female cancers from 2022 to 2040. DALYs, disability-adjusted life-years.
